# Virtual Reality Applications for Neurological Disease: A Review

**DOI:** 10.3389/frobt.2019.00100

**Published:** 2019-10-16

**Authors:** Eirini Schiza, Maria Matsangidou, Kleanthis Neokleous, Constantinos S. Pattichis

**Affiliations:** ^1^Research Centre on Interactive Media, Smart Systems and Emerging Technologies (RISE), RISE Limited (RISE), Nicosia, Cyprus; ^2^eHealth Laboratory, Department of Computer Science, University of Cyprus, Nicosia, Cyprus

**Keywords:** virtual reality, head-mounted display (HMD), fully-immersive systems, neurorehabilitation, review – systematic

## Abstract

Recent advancements in Virtual Reality (VR) immersive technologies provide new tools for the development of novel and promising applications for neurological rehabilitation. The purpose of this paper is to review the emerging VR applications developed for the evaluation and treatment of patients with neurological diseases. We start by discussing the impact of novel VR tasks that encourage and facilitate the patient's empowerment and involvement in the rehabilitation process. Then, a systematic review was carried out on six well-known electronic libraries using the terms: “Virtual Reality AND Neurorehabilitation,” or “Head Mounted Display AND Neurorehabilitation.” This review focused on fully-immersive VR systems for which 12 relevant studies published in the time span of the last five years (from 2014 to 2019) were identified. Overall, this review paper examined the use of VR in certain neurological conditions such as dementia, stroke, spinal cord injury, Parkinson's, and multiple sclerosis. Most of the studies reveal positive results suggesting that VR is a feasible and effective tool in the treatment of neurological disorders. In addition, the finding of this systematic literature review suggested that low-cost, immersive VR technologies can prove to be effective for clinical rehabilitation in healthcare, and home-based setting with practical implications and uses. The development of VR technologies in recent years has resulted in more accessible and affordable solutions that can still provide promising results. Concluding, VR and interactive devices resulted in the development of holistic, portable, accessible, and usable systems for certain neurological disease interventions. It is expected that emerging VR technologies and tools will further facilitate the development of state of the art applications in the future, exerting a significant impact on the wellbeing of the patient.

## Introduction

In recent years, Virtual reality (VR) technology has gained recognition as a useful tool for cognitive research, evaluation, and rehabilitation. A relatively new and a less explored area of VR applications is rehabilitation, helping patients who have lost some of their physical, and/or cognitive abilities to regain these. VR systems allow users to interact in various sensory environments and to obtain real-time feedback on their performance without exposing them to risks while using computer technology. The simulated environments offered via VR technology make it possible for patients to participate in activities in settings and environments like those encountered in real life. In addition, VR tools can be used to record accurate measurements of the user performance and to deliver greater therapeutic stimulation to users.

Some VR applications used in healthcare are for easing pain, anxiety, and distraction where the patient can find himself in an environment of their preference. These applications can provide better mental health and finer quality of life to the patient. Other VR applications are used for cognitive training and patient can work their cognitive abilities by playing a game, while also integrating physical excise aspects. Finally, one of the most complex application solutions in healthcare with VR are physical and neurological rehabilitation. These applications provide functional goals programmed into the virtual reality interactive games, and patients will be able to have a much more fun and engaging therapy experience that will help them rebuild their neurological pathways and inevitably give them the exercise and workout they need. Some examples of these applications can be driving assessment after brain injury where the patient tries to regain his ability to drive. This example can help the patient for his cognitive, motor, and sensory factors. Another common application is the virtual classroom scenario which consists a standard rectangular classroom environment containing desks, a teacher, a blackboard, a side wall with large windows etc. Within this scenario, children's attention performance can be assessed while a series of typical classroom distracters are systematically controlled and manipulated within the Virtual Environment (VE) (Weber, 2019).

Although the use of VR applications is increasing, to the best of our knowledge no systematic review has investigated the use of consumer-oriented fully-immersive VR applications in neurorehabilitation in the past few years along with their effect of these on cognition. To address this gap, the present review examines emerging VR applications developed for the evaluation and intervention of patients suffering from certain neurological diseases.

There are three types of VR systems (Ma and Zheng, 2011): (i) Non-immersive VR systems, is a desktop computer based 3D graphical system which allows the user to navigate the VE that is displayed on a computer screen, typically with the keyboard and the mouse; (ii) Semi-immersive systems project the graphical display onto a large screen, and may rely on some forms of gesture recognition system to implement more natural interactions; (iii) Fully-immersive systems in which the users' vision is fully enveloped, creating a sense of full immersion via a head-mounted display (HMD).

**Figure 1:**
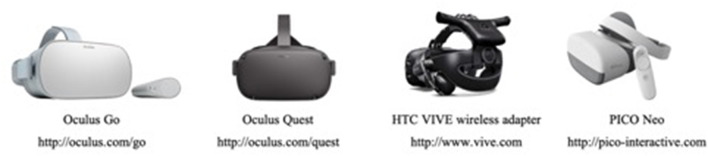
Selected VR HMDs from left to right, the Oculus GO, Oculus Quest, HTC VIVE wireless adapter, and PICO Neo.

Consumer-oriented fully-immersive VR technologies have advanced quite significantly in the past five years (Table 1). These new affordable immersive VR technologies could provide an ideal solution for real clinical settings (Anthes et al., 2016 and Matsangidou et al., 2017). Affordable hardware and open source software prescribe the resources needed to introduce new VR applications. These concepts have successfully managed to address past problems and limitations especially regarding the level of immersiveness and user's interaction in VR applications (Figure 1).

**Table 1:** Selected VR technologies and indicative costing according to Amazon accessed September 2019.

**VR technology**	**Release date**	**Cost**	**Company**	**Website**
Google cardboard	25/06/2014	$5.71–$39.95	Google, US	https://vr.google.com/cardboard/get-cardboard/
Oculus gear VR	27/11/2015	$129.99	Oculus, US	https://www.oculus.com/gear-vr/
Oculus rift	28/03/2016	$399	Oculus, US	www.oculus.com/en-us/rift/
HTC vive	05/04/2016	$599–$1199	HTC, US	www.htcvive.com
Sony playstation	13/10/2016	$469.95–$549.95	Sony, AU	www.playstation.com/en-au/explore/playstation-vr/
Oculus GO	06/12/2016	$199–$249	Oculus, US	https://www.oculus.com/go/

Wireless HMDs, haptic input devices, virtual sensory vests omnidirectional treadmills, accurate, and precise tracking systems and optical scanners for gesture-based interaction are nowadays considered to be among the most prominent trends in the field of VR (Anthes et al., 2016). Importantly, most of these technologies incorporate precise and robust sensory data acquisition that can be used in a wide range of applications including medicine, sports training, education, and physical/mental rehabilitation.

The objective of this paper was to carry out a systematic review of emerging VR applications developed over the last 5 years, covering selected neurological diseases. More specifically, this review paper covers the following diseases: dementia, stroke, spinal cord injury, Parkinson's, and multiple sclerosis. The paper is organized as follows. Section Literature Review Method covers the literature review methodology in neurological disorders. Section Review of VR Studies in Neurological Diseases presents the results of the literature review and discusses the findings under the following three subsections: the effectiveness of VR in neurorehabilitation, Virtual Environments (VE), VR and interactivity devices, and intervention strategies and system evaluation. Section Emerging Technologies covers briefly the VR emerging technologies and the introduction of intelligent decision making and adaptive feedback in forthcoming VR applications. Finally, section Concluding Remarks provides some concluding remarks of the study.

## Literature Review Method

The review was conducted based on Bargas-Avila and Hornbæk (2011) and Cochrane methodologies (Khan et al., 2001; Deeks et al., 2008), which consisted of the following five phases.

### Procedure

#### Phase 1: Potentially Relevant Publications Identified

##### Electronic Libraries

We searched six electronic libraries, to cover a balanced range of disciplines, including computer science/engineering, medical research, and multidisciplinary sources. The libraries which included in the review were:

1. ACM Digital Library (ACM)
2. Google Scholar
3. IEEE Xplore (IEEE)
4. MEDLINE
5. PubMed
6. ScienceDirect (SD).

We restricted the search to a timeframe of five years (2014–2019), since we are aiming in only in fully immersive VR technologies have emerged for consumer use during this time (see examples given in Table 1).

##### Search terms

Our aim was to search for neurorehabilitation techniques that use immersive VR technology. Therefore, we have used the following two queries exactly to the aforementioned six libraries:

– Virtual Reality AND Neurorehabilitation
– Head Mounted Display AND Neurorehabilitation.

##### Search procedure

The above terms were searched in the following fields: full text (if available), title, abstract and keywords.

##### Search results

The total search that returned in phase 1 can be seen in Table 2. At the end of this phase, all corresponding PDFs were downloaded for the analysis to be conducted.

**Table 2:** Number of publications identified per library.

	**ACM**	**Google scholar**	**IEEE**	**MEDLINE**	**PubMed**	***SD***
Virtual reality AND neurorehabilitation	39	172	115	3	335	220
Head mounted display AND Neurorehabilitation	24	0	112	0	11	63
Total findings	1,094					

#### Phase 2: Publications Retrieved for Detailed Evaluation

##### First exclusion

A total of 1,069 articles were further analyzed after excluding manually 25 articles with wrong years entries.

##### Second exclusion

Duplicate publications across libraries (e.g., different libraries producing the same result) and *within* each library (e.g., different terms producing the same result within the same library) were removed.

We removed 32 duplicate publications across libraries, ending up with 1,047 different articles. After removing 36 duplicates *within* each library we ended up with 1,001 different articles.

##### Third exclusion

We narrowed the entries down to the original full articles that were written in English. We excluded 645 articles that we did not have access to the full length, 46 review articles, 37 articles that were not in English, and 18 articles that were not full peer-reviewed articles (e.g., referred to workshops, posters, presentations, magazine articles, theses). With these criteria, we excluded 746 articles. The remaining 255 articles comprised of journal and conference articles.

#### Phase 3: Publications to Be Included in the Analysis

##### Final exclusion

**Figure 2:**
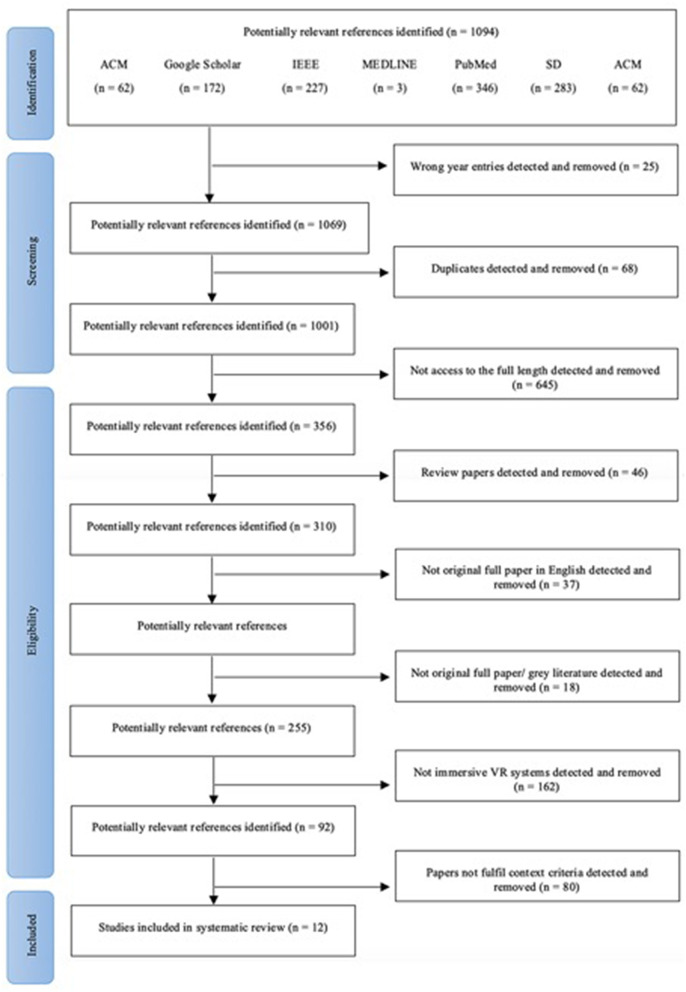
Article identification and selection flow diagram.

The focus on this review was placed on fully-immersive VR systems, therefore we excluded articles which used non-immersive or semi-immersive VR systems. Based on these criteria, we excluded 163 further articles which did not use fully-immersive VR technology and 8 articles that did not specify the type of VR equipment. We also excluded 24 articles that were not relevant to a nervous system injury linked to functional disability. Finally, we excluded 48 irrelevant articles that appeared in the first phase and were not excluded during the second phase filtering. These articles appeared in our search because they contain relevant words to the ones that we searched for, but did not match with the specific technology content. Based on these restrictions, in this phase we removed 240 irrelevant publications. As a result, we ended up with 12 relevant articles (10 journal articles and, 2 conference articles) (Figure 2).

#### Phase 4: Data Collection

In this phase, we extracted all the relevant information from the articles for the analysis to be conducted. Specifically, for each study, we recorded the objectives, the sample size, the condition or the population characteristics, the content of the VEs used, the interactivity devices used, the methodology/interventions the study was based on, other instruments used and the key findings. Moreover, we labeled each study, based on the result as positive (+), negative (-), or neutral ().

#### Phase 5: Data Analysis

Descriptive statistics were used to characterize the data from Phase 4. Thematic analysis was used as well to categorize our findings in themes, i.e., the population's characteristics, the types of the VEs, the interactivity devices used in the study, and the key findings. Inter-coder reliability was carried out to determine the correspondence of coding across researchers (between first and second author). Using the Cohen's Kappa formula, a reliability of 0.81 was computed.

## Review of VR Studies in Neurological Diseases

All 12 studies examined the use of VR in samples with conditions of a nervous system injury linked to functional disability. In particular, most of the studies examined the use of VR for people living with dementia (PwD) (*n* = 4), stroke (*n* = 3), spinal cord injury (*n* = 2), parkinson's (*n* = 1), multiple sclerosis (*n* = 1), and phantom upper limb pain (*n* = 1). Table 3 presents the sample size and the participant characteristics for each study.

**Table 3:** Sample size/participants characteristics.

**Study**	**Sample**	**Participant characteristics**
**Dementia**		
Hodge et al., 2018	7	Dementia: 4 PwD; 3 Family Members
Mendez et al., 2015	5	Dementia
Rose et al., 2019	24	Dementia: 8 PwD; 16 Caregivers
Tabbaa et al., 2019	24	Dementia: 8 PwD; 16 Caregivers
**Multiple Sclerosis**		
Peruzzi et al., 2016	8	Multiple sclerosis
**Parkinson**		
Kim et al., 2017	33	Parkinson: 11 PD; 11 Healthy Young Adults; 11 Healthy Older Adults
**Stroke**		
Gamito et al., 2017	20	Stroke
Saleh et al., 2017	14	Stroke
Standen et al., 2017	27	Stroke: Arm dysfunction
**Spinal Cord Injury**		
Donati et al., 2016	8	Spinal cord injury
Pozeg et al., 2017	40	Spinal cord injury: 20 SCI; 20 Healthy—Control
**Phantom Upper Limb Pain**		
Ichinose et al., 2017	9	Phantom upper limb pain

### The Effectiveness of Virtual Reality in Neuro-Rehabilitation

Overall, VR seems to show a promising potential for Neuro-Rehabilitation (Table 4). Ten out of 12 studies illustrated positive outcomes in the use of VR for the treatment of nervous system injury linked to functional disability. While the other two outlined the opportunities and challenges inherent to the design and use of VR with people with dementia and their careers (Hodge et al., 2018), and they used VR only as a tool to support the intervention for the treatment of stroke (Saleh et al., 2017).

**Table 4:** VR effectiveness.

**Study**	**Objectives**	**Results**	**Label**
**Dementia**			
Hodge et al., 2018	(1) Design VR experiences for PwD; (2) Explore the reactions of PwD to VR; (3) Design a personalized experience.	Outline opportunities and challenges are inherent to the design and use of VR experiences with people with dementia and their careers.	()
Mendez et al., 2015	Assess the feasibility of VR and VR-Socialization for PwD.	(1) No adverse effects reported; (2) High rates of presence reported; (3) PwD tended to the greater verbal elaboration of answers in VR compared to real-world interviews.	(+)
Rose et al., 2019	Feasibility of VR for PwD.	(1) VR was tried and accepted by PwD; (2) PwD viewed VR as a ‘change in the environment’ and would use it again; (3) PwD experienced pleasure during and after VR and increased alertness because of VR; (4) Findings evidenced the clinical feasibility of VR for PwD.	(+)
Tabbaa et al., 2019	(1) Discuss the appeal and the impact of VR for PWD; (2) Present VR design opportunities, pitfalls, and recommendations for future deployment in healthcare services; (3) Demonstrate the potential of VR for PWD in locked settings.	VR is a feasible solution for PWD in long-term care.	(+)
**Multiple Sclerosis**			
Peruzzi et al., 2016	Assess the feasibility of VR treadmill for MS.	(1) Gait speed and stride length improved; (2) The ability to overcome obstacles was improved; (3) VR treadmill is feasible and safe for MS.	(+)
**Parkinson**			
Kim et al., 2017	Evaluate the safety of using VR for longer bouts of walking for individuals with PD.	(1) No adverse effects reported; (2) Lower Stress levels reported; (3) PD patients can successfully use VR during walking.	(+)
**Stroke**			
Gamito et al., 2017	Test the effectiveness of a VR for neuropsychological rehabilitation, a cognitive training program.	(1) Significant improvements in attention and memory functions; (2) The findings provide support for the use of VR cognitive training in neuropsychological rehabilitation.	(+)
Saleh et al., 2017	Test the interactions between regions in the brain that may be important for modulating the activation of the ipsilesional motor cortex during MVF.	Significant mirror feedback modulation of the ipsilesional motor cortex arising from the contralesional parietal cortex, in a region along the rostral extent of the intraparietal sulcus.	()
Standen et al., 2017	Feasibility of home-based VR of arm rehabilitation following stroke.	Significant improvement in the final Motor Activity Log.	(+)
**Spinal Cord Injury**			
Donati et al., 2016	Investigate the clinical impact of the Walk Again Rehabilitation, based on VR BMI.	(1) Neurological improvements in somatic sensation; (2) Regained voluntary motor control in key muscles; (3) Improvement in walking index; (4) 50% of patients upgraded to paraplegia classification.	(+)
Pozeg et al., 2017	Investigate changes in body ownership and chronic neuropathic pain in SCI using VR.	(1) SCI is less sensitive to multisensory stimulations inducing illusory leg ownership (2) Leg ownership decreased with time for SCI. (3) No differences between groups in global body ownership detected.	()
**Phantom Upper Limb Pain**			
Ichinose et al., 2017	Investigate the analgesic effect produced by tactile feedback using visual feedback.	(1) The pain was significantly lower during the VR Condition; (2) VR somatosensory feedback can improve the analgesic effect of the affected limb.	(+)

Detailed analysis of the studies revealed that specific characteristics of the population, such as the type of disease, influence the study objectives, and the outcomes. With respect to the four studies of dementia, it was shown that all the studied objectives examined the feasibility of VR for people living with dementia (4/4). The feasibility of VR technology for people with dementia was examined with two different approaches. Two out of four studies (Hodge et al., 2018; Tabbaa et al., 2019) evaluated the technology feasibility from a patient-centered designed perspective targeting a human-computer interaction audience, whereas the rest of the studies adopted a psychology/psychiatric perspective to evaluate VR's feasibility (Mendez et al., 2015; Rose et al., 2019). All studies concluded that findings evidenced the clinical feasibility of VR for people with several stages of dementia. No adverse effects were stated, and high rates of pretense/immersion and positive emotional responses were reported.

Dementia was not the only disease that studies examined the feasibility of VR. From the review, it was found that multiple stroke (Standen et al., 2017), Parkinson (Kim et al., 2017), and sclerosis (Peruzzi et al., 2016) diseases were also linked to feasibility studies of VR. The results were in line with dementia studies. Importantly the VR's effectiveness was further enhanced by a study that examined the feasibility of long term (8 weeks) home-based VR of arm rehabilitation following stroke indicating that VR can be used as a personalized solution in home-based contexts (Standen et al., 2017).

VR was also used for neuropsychological rehabilitation based on a cognitive training program for stroke patients (Gamito et al., 2017). The results suggested that VR can be used as a cognitive training tool illustrating significant improvements in attention and memory functions. VR was also tested as a walk again rehabilitation tool for spinal cord injury patients. It demonstrated significant regain in voluntary motor control which resulted in walking improvements (Donati et al., 2016).

Finally, VR revealed promises in response to the treatment of phantom limb pain, since it was shown that tactile feedback via VR visual feedback was able to diminish pain and improve the analgesic effect of the affected limb (Ichinose et al., 2017).

### Virtual Environments, Virtual Reality, and Interactivity Devices

The VR devices used for the treatment of nervous system injury linked to functional disabilities were eMagin Z800 (3/12), Google Cardboard (3/12), and Oculus Rift (3/12). The rest of the studies did not specify the VR equipment (3/12). Almost half of the studies (5/12) did not use any interactivity equipment and they used VR only to transport the patient into a different environment. Two studies used a Virtual Glove as interactivity device and the rest of the studies (5/12) used Xsens sensors, Vizard, Keyboard, EEG-based BMI, and Kinect to allow the user to interact with the VE.

From the analysis it was derived that most of the dementia studies used a Google Cardboard (3/4) (Hodge et al., 2018; Rose et al., 2019; Tabbaa et al., 2019) and an eMagin Z800 (1/4) (Mendez et al., 2015) VR device with no interactivity sensors (4/4). Simple VEs with natural scenes were used by most of the studies (3/4). Based on these findings (Table 5) we can conclude that VR's feasibility for people with dementia does not require any expensive VR equipment and interactivity devices.

**Table 5:** Virtual reality, interactivity devices and content.

**Study**	**Virtual environments**	**VR device**	**Interactivity devices**
**Dementia**			
Hodge et al., 2018	(1) A simple apartment, which allowed participants to turn their head and see out of a window; (2) A park, based on a local park in the area; (3) A tropical beach with a horse running along the sand.	Google cardboard	No
Mendez et al., 2015	The PwD was seated in a chair at the end of the conference table and told that they would be interviewed by the five avatars. They were asked to answer their questions as if they were real people. The avatars asked a series of questions.	eMagin Z800	No
Rose et al., 2019	(1) Cathedral; (2) Forest; (3) Sandy beach; (4) Rocky beach; (5) Countryside.	Google cardboard	No
Tabbaa et al., 2019	(1) Cathedral; (2) Forest; (3) Sandy beach; (4) Rocky beach; (5) Countryside.	Google cardboard	No
**Multiple Sclerosis**			
Peruzzi et al., 2016	A tree-lined trail with obstacles to appear on the trail.	eMagin Z800	Xsens
**Parkinson**			
Kim et al., 2017	A cityscape with buildings, animated avatars, and a straight sidewalk. Participants were able to freely look around the scene while walking.	Oculus rift	Vizard
**Stroke**			
Gamito et al., 2017	Several daily life activities: (1) Buy several items; (2) Find the way to the minimarket; (3) Find a virtual character dressed in yellow; (4) Recognize outdoor advertisements; (5) Digit retention.	eMagin Z800	Keyboard
Saleh et al., 2017	Hand mirror visual feedback in VR.	Not stated	Virtual glove
Standen et al., 2017	(1) Space-race: Pronation and supination of the hand to guide a spacecraft through obstacles; (2) Sponge-ball: Open their fist and extend their fingers to release a ball to hit a target. (3) Balloon-pop: Balloon was grasped and popped by moving it to a pin.	Not stated	Virtual glove
**Spinal Cord Injury**			
Donati et al., 2016	A 1st person's perspective virtual avatar body with rich visual and tactile feedback.	Oculus rift	EEG-based BMI
Pozeg et al., 2017	Virtual Avatar as a 3rd person perspective.	Not stated	No
**Phantom Upper Limb Pain**			
Ichinose et al., 2017	Repeatedly touched a target object with the affected limb, by converting via Mirror Visual Feedback the movements of the intact limb.	Oculus rift	Kinect

Patients with Parkinson (Kim et al., 2017) and multiple sclerosis (Peruzzi et al., 2016) were assigned to use Oculus Rift and eMagin Z800 VR devices paired with Xsens and Vizard sensors respectively. Both studies simulated walking VEs. A study with spinal cord injury patients (Donati et al., 2016) also used walking VEs based on EEG-based BMI interactivity device and an Oculus Rift HMD.

Two studies, with stroke (Saleh et al., 2017) and Phantom Limb pain Patients (Ichinose et al., 2017) used VR Oculus rift paired with Cyberlove and Kinect sensors, as an alternative solution to Mirror Box therapy. In mirror box therapy the patient was instructed to be seated in front of a mirror. The mirror's orientation was parallel to the patient's midline. At this position, the patient could see through the mirror the reflection of his/her unaffected body part. The affected body part was hidden beside the mirror and under the mirror box. This position created the visual illusion that the affected body part is working properly since visual cues were created through the mirror and from the opposite side of the unaffected body part in response to the brain's commands (Ramachandran, 2005). VR replicated the traditional mirror box in a technologically advanced version. More specifically, the mirror box was replaced by the VE and sensors to reproduce the movements of the unaffected body part. To conclude, the type of disease affects the selection of VEs, the VR and the interactivity devices.

### Intervention Strategies and System Evaluation

The intervention strategies were divided in: (i) single testing, where the patient was exposed to the VR system only once, and (ii) multiple testings' where the patient used of the system for a long period of time incorporated into the rehabilitation training (i.e., from 6 weeks or up to a year) (Table 6).

**Table 6:** Intervention strategies and system evaluation materials.

**Study**	**Intervention Strategies**	**Evaluation Materials**
**Dementia**		
Hodge et al., 2018	Single Intervention: VR Experiencing and co-design testing.	(1) Field notes; (2) Audio recordings; (3) Interviews.
Mendez et al., 2015	Single Intervention: PwD answered questions that were given by avatars.	(1) Interviews by VR avatars; (2) Heart Rate; (3) Self-reports: Arousal, Stress, Anxiety, Anger, Fatigue, Attention; (4) Interviews; (5) University of California at Los Angeles Structured Insight Interview; (6) Emotional Insight; (7) Mini-Mental State Examination; (8) Clinical Dementia Rating Scale; (9) Functional Activities Questionnaire; (10) Frontal Assessment Battery; (11) Frontal Systems Behavior Scale; (12) Wisconsin Card Sort Test.
Rose et al., 2019	Single Intervention: VR exposure as feasibility testing.	(1) Overt Aggression Scale-Modified for Neurorehabilitation; (2) St Andrews Sexual Behavior Assessment; (3) Observed Emotion Rating Scale; (4) Time; (5) Semi-structured Interviews.
Tabbaa et al., 2019	Single Intervention: VR exposure as feasibility testing.	(1) Overt Aggression Scale-Modified for Neurorehabilitation; (2) Observed Emotion Rating Scale; (3) Semi-structured Interviews (based on the System Usability Scale, Presence); (4) Observations.
**Multiple Sclerosis**		
Peruzzi et al., 2016	Six Weeks Training: Subjects were asked to walk over-ground in the gait analysis laboratory under two conditions: (a) at comfortable speed; (b) while serially subtracting the number “3” from a predefined 3-digit number.	Pre, Post, and Follow-up: (1) Collect Marker Trajectories and Ground Reaction Forces; (2) Joint kinematic Parameters (peak values of the kinematic curves); (3) Kinetic Parameters (maximum values of the joint moments and power during gait phases); (4) Six-minute Walk Test; (5) Square Step Test; (6) Expanded Disability Status Scale.
**Parkinson**		
Kim et al., 2017	Single Intervention: VR exposure of four bouts of 5 min walking to assess the feasibility of the VR walking.	(1) Movement Disorder Society Unified Parkinson's Disease Rating Scale; (2) Self-Selected Walking Speed; (3) Mini-Balance Evaluation Systems Test; (4) 14-item Balance Assessment for Dynamic Balance and Gait; (5) Activities-Specific Balance Confidence; (6) Center of pressure; (7) Simulator sickness questionnaire; (8) Stress Arousal Checklist; (9) Presence.
**Stroke**		
Gamito et al., 2017	Six Weeks Training: Randomly divided into 2 conditions: (1) VR 60 cognitive stimulation; (2) control waiting list.	(1) Wechsler Memory Scale; (2) Toulouse–Pieron Test; (3) Rey Complex Figure.
Saleh et al., 2017	Single Intervention: A VR goal-directed finger flexion movement with their unaffected hand while observing real-time visual feedback of the corresponding (veridical) or opposite (mirror) hand.	fMRI
Standen et al., 2017	Eight Weeks Training: Randomly divided into 2 conditions: (1) VR employing infrared capture to translate the position of the hand into gameplay or usual care; (2) Control - usual care.	(1) Wolf Motor Function Test; (2) Nine-Hole Peg Test; (3) Motor Activity Log; (4) Nottingham Extended Activities of Daily Living.
**Spinal Cord Injury**		
Donati et al., 2016	12 Months Training: (1) an immersive virtual reality environment in which a seated patient employed his/her brain activity, recorded via a 16-channel EEG, to control the movements of a human body avatar, while receiving visuotactile feedback; (2) identical interaction with the same virtual environment and BMI protocol while patients were upright, supported by a stand-in-table device; (3) training on a robotic body weight support (BWS) gait system on a treadmill; (4) training with a BWS gait system fixed on an overground track; (5) training with a brain-controlled robotic BWS gait system on a treadmill; (6) gait training with a brain-controlled, sensorized 12 degrees of freedom robotic exoskeleton. Clinical evaluation started on the first-day patients began training (Day 0) and was repeated after 4, 7, 10, and 12 months.	(1) American Spinal Injury Association; (2) Impairment Scale; (3) Semmes-Weinstein Monofilament Test; (4) Temperature Evaluation; (5) Lokomat L-force Evaluation; (6) Thoracic-Lumbar Scale; (7) Walking Index Spinal Cord Injury II; (8) Spinal Cord Independence Measurement III; (9) McGill Pain Questionnaire; (10) Visual Analog Scale; (11) Medical Research Council scale; (12) Modified Ashworth Scale; (13) Lokomat L-stiff Evaluation for spasticity; (14) World Health Organization Quality of Life Assessment Instrument-Bref; (15) Rosenberg Self-Esteem Scale; (16) Beck Depression Inventory.
Pozeg et al., 2017	Single Intervention: 2 × 2 repeated measures design, we manipulated the synchrony between the stroking of the virtual legs (synchronous/asynchronous) and the participant's back location (lower/upper back). In the synchronous condition, the stroking of the virtual legs was synchronized with the stroking of the participant's back. In the asynchronous condition, the visuotactile stimulation was delayed 1 s.	Questionnaires: (1) Body Illusions Studies; (2) Body ownership; (3) Visual Analog Scale; (4) Cambridge Depersonalization Scale.
**Phantom Upper Limb Pain**		
Ichinose et al., 2017	Single Intervention: Randomly divided in 3 conditions: (1) VR—applied tactile feedback to their cheek when their virtual affected limb touched a virtual object; (2) Control A—tactile feedback was either applied to their intact hand (Intact Hand Condition); (2) Control B—Not applied at all (No Stimulus Condition).	Pre and Post: McGill Pain Questionnaire.

**Figure 3:**
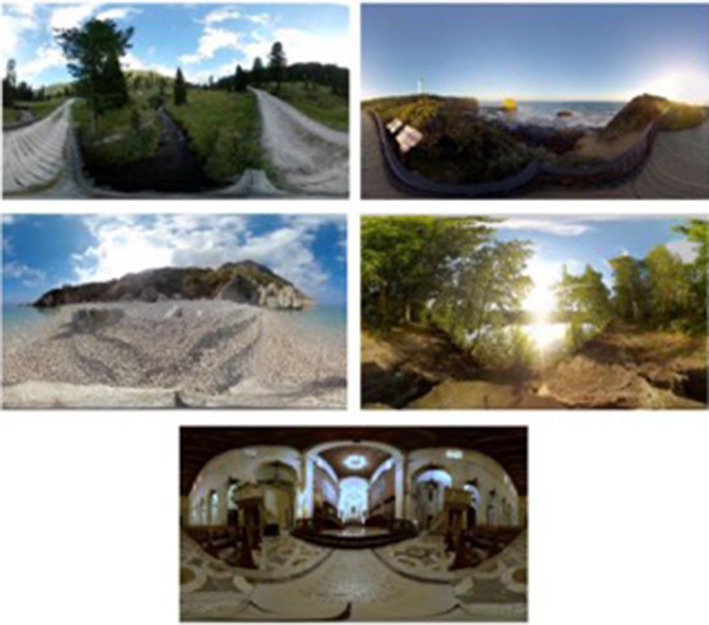
Actual figure from Rose et al. (2019) paper, presenting the five options of VR environments given to patients with dementia.

In the aforementioned studies, dealing with people living with dementia, the feasibility of VR technology (3/4) was tested only once. Therefore, the intervention strategies were mostly associated with the development and the design of the technology from a patient-centered perspective (Hodge et al., 2018; Rose et al., 2019; Tabbaa et al., 2019). In particular, researchers along with clinical staff (Rose et al., 2019; Tabbaa et al., 2019) and patients with dementia (Hodge et al., 2018) designed a VR system responsible to expose the patient into a different environment. All four studies used observation notes along with interview materials to evaluate the effectiveness of the system. Quantitative scales, such as arousal, stress, anxiety, anger, fatigue, and attention self-reports were also used to enhance the qualitative data (Figure 3).

The feasibility of VR was also tested for older adults with parkinson's enhanced by a walking task on a treadmill (Kim et al., 2017). Thirty-three participants (11 healthy young, 11 healthy older adults, and 11 individuals with PD) were recruited for this study and assigned to a 20 min walking tasks on a treadmill while watching a virtual city scene. Comparisons were made between the three different populations.

**Figure 4:**
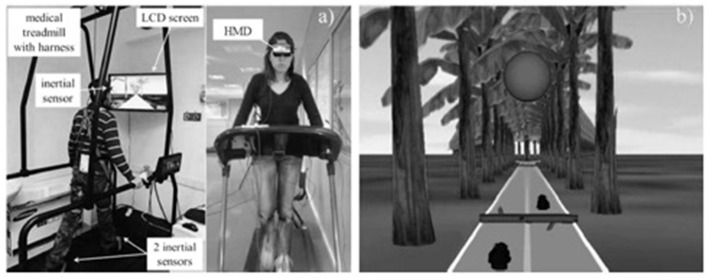
Actual figure from Peruzzi et al. (2016) paper, presenting **(a)** The experimental set-up; **(b)** The virtual environment.

Patients with multiple sclerosis were asked to perform walking tasks on a treadmill watching a VR environment representing a tree-lined trail under a comfortable speed (Peruzzi et al., 2016). They were also asked to perform another walking task while serially subtracting the number “3” from a predefined 3-digit number. During the intervention, patients were required to pass obstacles aerating on the trail, while several dynamic distractors were also added to the VE to challenge the patient's attention. Each patient used a personalized environment based on personal gait problems (i.e., decreased foot clearance, obstacle avoidance, and problems with planning). Successful and unsuccessful passes, as determined by the inertial measurements, were rendered to the subject during the trial with visual and auditory feedback. A cognitive concurrent task was added by asking the subject to memorize the route to follow, which was shown to them prior to the trial. The training lasted for 6 weeks with each session to last about 45 min, with pre, post and follow-up materials to assess walking endurance and obstacle negotiation (Figure 4).

**Figure 5:**
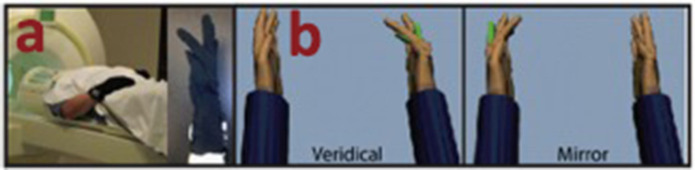
Actual figure from Saleh et al. (2017) paper, presenting **(a)** the experimental set-up and equipment; **(b)** the virtual mirror feedback.

Apart from VR for walking tasks, interventions were also focused on affected upper limb training for patients dealing with stroke and phantom limb pain. In particular, Saleh et al. (2017) evaluated the effectiveness of VR with mirror visual feedback as a single intervention with the aim to facilitate recovery of disordered movement and stimulate activation of under-active brain areas due to stroke. During the experiment, patients were instructed to move the non-paretic hand's finger and watched the back-projected visual stimuli reflected in a mirror within the VR environment. The finger motion was back-projected onto a screen, showing two virtual hand models. On a given trial, the motion of the unaffected hand actuated one of the VR hands, located on the same (Veridical), or opposite (Mirror) side relative to the actual hand. The “move” prompt was displayed for the duration of the trial event (5s), and the “rest” prompt was displayed for the duration of the rest period (random 4–7-sec jittered). Subjects were instructed to complete the movement within the “move” epoch. Each scanning run included eight repetitions of four randomly interleaved visual feedback conditions and evaluated based on brain scanning reports. Similarly, mirror visual feedback was also used for phantom limb pain. Patients were instructed to touch via VR a virtual target. Once again during the experimental condition patients were instructed to move the non-affected hand to touch the virtual target and watched back in the VR the affected hand to perform the task. Pre and Post pain scales were used to evaluate the effectiveness of the system (Figure 5).

**Figure 6:**
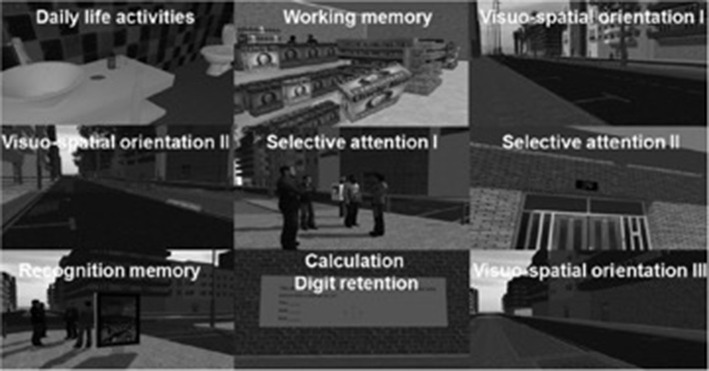
Actual figure from Gamito et al. (2017) paper, presenting the nine virtual cognitive trainings.

Finally, cognitive training intervention was also used via VR for the treatment of stroke (Gamito et al., 2017). The VR system was developed based on a serious games application for cognitive training, enhanced with attention and memory tasks consisting of daily life activities. The cognitive training VR scenarios were invented to train cognitive functions such as working memory tasks (i.e., buying several items), visuospatial orientation tasks (i.e., finding the way to the minimarket), and selective attention tasks (i.e., finding a virtual character dressed in yellow), recognition memory tasks (i.e., recognition of outdoor advertisements) and calculation (i.e., digit retention). Twenty stroke patients were randomly assigned to two conditions: exposure to the intervention and waiting list control to evaluate the effectiveness of using VR for cognitive training. Several scales were used to identify the effectiveness of the system (Figure 6).

## Emerging Technologies

### Virtual Reality Input and Output Devices

New and emerging hardware developments are not yet commercially available. However, it is still possible to identify the technological trends particularly under the two main VR categories of input and output devices.

*Input devices* mostly refer to the controllers that are often enhanced by haptic feedback and hand and body tracking. A second input category is the navigation devices that bring to the user the illusion of moving through endless spaces within VEs such as one-direction and omnidirectional treadmills and passive low-friction surfaces, or “slidemills.” Slidemill refers to devices like treadmills with the difference that the surface under the user's foot is static, therefore, the interface feels less natural and thus less immersive. Another form of input tracking system is hand and body tracking devices. User's posture estimation using inertial measurement units (IMUs) combined with magnetic tracking can be used to provide a reasonable self-representation in HMDs that elevates the feeling of realism in VEs. Finally, gesture tracking devices range from data gloves, with strain gauges or fiber optics that are often used combined with technologies using optical tracking and electromyography (EMG) signals that capture wrist movements with very promising prospects for VR applications in different fields especially for physical and cognitive rehabilitation.

*Output devices* primarily focus on the visual displays or more precisely wired or mobile HMDs when considering the VR field.

Wired HMDs specifications concentrate on quality factors like resolution, Field of View (FOV) or weight. Some wired HMDs are equipped with cameras for Augmented Reality (AR) applications and can be used as video see-through displays. Recently, the tendency in large VR companies is to include also eye tracking in the visual displays (e.g., Tobii VR^1^, Steam FOVE^2^, and SMI Eye tracking^3^).

On the other hand, mobile HMD systems run the applications wirelessly and without the need to be connected to a PC. Usually, these systems rely on smartphone technologies combined with ergonomically designed smartphone cases for stand-alone systems. Some examples of such standalone systems that have been released since 2018 include the Oculus Go^4^, Oculus Quest^5^, HTC VIVE focus^6^, Pico Neo^7^, and Xiaomi MI VR^8^. In addition to the later standalone systems, some manufacturers designed mobile devices with the option to use wireless adaptors for remote connection of the HMDs with PCs that run the VR applications (e.g., the HTC VIVE wireless adapter option^9^). Another important category of the output device are systems that include haptic and multi-sensory feedback. Haptic devices usually focus on a different sensory system with approaches that exist in the form of vests including Vibro-tactile elements. Ubiquitous displays providing sensory haptic feedback has also been undertaken like for instance, the example of viewing the effort to develop a system that generates airflow around the user to simulate weather conditions based on the application that the user is experiencing.

Other multisensory displays include head-mounted masks with the ability to produce different scents to further increase the feeling of immersion to the user as it was described by (Badler et al., 1992). Examples for multisensory devices involve integrated systems that blow cool and warm air in the users face or even combine ultrasonic ionizing systems that generate water mist (Matsukura et al., 2011). In addition, significant scientific research is being published with respect to olfactory information integrated into VR displays to increase the user's sense of presence in VR (Chen, 2006; Nakaizumi et al., 2006).

### Intelligent Systems and Adaptive Feedback

Adaptation in a system involves a set of interacting entities that together can respond to changes and usually includes processing of feedback information from the output of the system to readjust the states of the system in a next time instance forming what is as “controlled close loops.” Control loops in adaptive systems and machine learning are mostly used for prediction, recognition, detection, and optimization (Vaughan et al., 2016).

A recent literature review regarding the integration of computational intelligence and adaptation with VR technologies clearly demonstrated the prospects of achieving high impact results when combining these elements in application areas such as medicine, education training and gaming (Vaughan et al., 2016). Especially in applications that require trainee-specific and individual adaptive content, automation, machine learning and data driven features can guide feedback information to the inputs of autonomous systems and build new and customized training sessions based on individual requirements (Vaughan et al., 2016).

Some examples of self-adaptive systems in VR applications include automatically generated haptic, visual and auditory feedback signals that are used to modify the virtual scenarios and trigger methods to adapt the environmental behavior (e.g., Luzanin and Plancak, 2014). In addition, sensory information from assessment and scoring mechanisms, objectively facilitate the design of more optimum setups with automatically generating user-centered content (Wanzel et al., 2002; Vaughan et al., 2015).

Considering the above, adaptation and machine learning elements in rehabilitation tasks are very well suited because of the need to engage users and to intelligently adapt exercises based on user's progress (Borghese et al., 2013; Pirovano et al., 2013). In addition, adaptive feedback in rehabilitation tasks can supplement the therapist's input with the creation of a self-learned virtual therapist (Kallmann et al., 2015). For example, Borghese et al. (2013) presented an intelligent adaptive solution with Bayesian networks and fuzzy systems based on Nintendo Kinect® motion sensing controllers for VR rehabilitation games (IGER) (Borghese et al., 2013).

Other examples include VR neurological rehabilitation systems that incorporate data mining of user scores and other measured performance data in a feedback computational intelligence loop to formulate a training plan for each trainee.

Future trends in virtual rehabilitation prescribe the path of new research for physiology driven adaptive VR systems this will allow the development of automated emotion recognition systems to be integrated in VR applications where the application responds appropriately to the emotions of their users (Popovic et al., 2009).

In addition, adaptive VR autonomous systems are currently enabling the performance of visio-haptic tasks without the requirement for human operator intervention. Accurate haptic simulation-based development platforms will inspire autonomous application with capabilities to convey the simulated VR information into a real-world haptic environment (like in surgery in autonomous neuro-rehabilitation tasks).

We consider that the technologies documented in this section will shape the development of the next generation of VR applications in rehabilitation. New virtual reality input devices will provide more complete data sets and signals about the behavior of the patient demanding intelligent processing, monitoring and profiling of the patient toward offering a personalized VR rehabilitation solution. Similarly, new output devices will facilitate VR applications to be more realistic, personalized and closer to the rehabilitation needs of the patient.

The aforementioned technologies will shape the development of state of the art VR rehabilitation services in the framework of emerging connected health systems and services (Pattichis and Panayides, 2019) in support of 4P's medicine (Golubnitschaja et al., 2016). More specifically, emerging VR applications will be (Golubnitschaja et al., 2016): (i) predictive: VR systems will automatically capture data to predict, manage, adapt, and/or deliver better treatment plans; (ii) pre-emptive: VR solutions will be designed to monitor vital signs and activities in real time which will communicate with personal health record archives and healthcare professionals; (iii) personalized interventions: new VR applications will enable the offering of best possible, most optimal, and innovative treatments; (iv) participatory: patient-centric VR applications will empower patients to be more active and allow the sharing of experiences. It is expected that emerging VR applications sharing the 4P's concept will trigger the offering of new services and business models for the benefit of the citizen.

## Concluding Remarks

Recent advances in VR immersive technologies provide new methods and tools for the development of novel and promising applications mainly for neurological rehabilitation. VR interventions have several advantages and are rapidly gaining ground as popular applications for different disease conditions. The big advantage of VR applications in rehabilitation is that they offer a “real-life like environment.” In addition, VR applications advantages include, control of stimulus presentation, and response measurements, safe assessment on different unsafe rehabilitation tasks, easy learning of the tasks to be performed, standardization of rehabilitation protocols, and enhanced user interaction and empowerment.

On the other hand, limitations of VR interventions include that the patient might forget that he/she is in a testing situation and the difficulty and complexity in generating personalized training environments. These prescribe some of the existing challenges to develop low-cost rehabilitation assessment and monitoring environments and applications. Furthermore, the development of VR technologies in recent years have resulted in more accessible and less expensive solutions, which could still provide positive results. However, the full potential of VR applications in healthcare still remains to be explored.

The purpose of this research work was to carry out a systematic review of emerging VR applications developed over the last 5 years, covering certain neurological diseases. Although, the final number of studies analyzed is rather small (12), still valuable input can be gained. It is expected, that the number of studies in consumer-oriented fully-immersive VR systems will significantly increase in the near future, given the rapid progression of development both in the hardware and software in these technologies.

The findings of this systematic literature review showed positive and promising results of using VR for rehabilitation exercise. It also suggests that low-cost, immersive VR technologies can prove to be effective for clinical rehabilitation in healthcare and home-based settings with practical implications and uses. Based on our review we found that dementia studies used the cheapest VR equipment (Goggles Cardboard) and no interactivity devices, achieving very good results. In addition, low-cost VR devices were found to be free of adverse effects, and high rates of presence/immersion, and positive emotional responses were reported. Consequently, it is now conceivable to use VR low-cost technologies with no interactivity devices to expose people with dementia in different environments, to improve pleasure and alertness. The application can evolve based on the needs and available budget one can have. It is also possible to experience VR outside of a specialized laboratory, making it more accessible to a wider group of patients if needed.

Even though most dementia studies used low-cost VR equipment with no interactivity devices, the rest of the studies apart from one (Spinal Cord Injury—Pozeg et al., 2017) used the following VR systems: Xsens, Vizard, EEG-based BM, Cyberlove, and Kinect sensors. These interactivity devices were responsible to transport the patients' movement into the VR environment in order to enhance the physical or cognitive training. VR and interactivity devices resulted in the development of a holistic portable, accessible and usable systems enabling the better handling of the neurological disorders reported. Furthermore, by employing machine learning and AI in VR applications, exercise interventions can be patient's specific to the treatment needs of the patient, thus, offering optimal care. Complex virtual therapy exercises need to be created with precise control over the stimulus and cognitive capacity that the user will experience.

Concluding, the main findings of this systematic literature review indicated that VR technology could be effective in improving the condition of the patient for certain neurological diseases. This review study outlined some key factors that may contribute to the effectiveness of VR applications, such as the objective of the study linked with the intervention strategy, the VR technology and interactivity equipment used in the study and other. It is expected that VR applications in healthcare will flourish within the next few years, triggering further investigations in different clinical settings. It is hoped that these VR applications could also prove to have an impact on the wellness of the patient that remains to be thoroughly investigated.

## Author Contributions

ES and MM conceived of the original idea. KN and CP supervised and assisted the findings of this work. All authors discussed the results and contributed to the final manuscript.

### Conflict of Interest

The authors declare that the research was conducted in the absence of any commercial or financial relationships that could be construed as a potential conflict of interest.

## Footnotes

^1^https://vr.tobii.com/

^2^https://www.getfove.com/

^3^https://www.smivision.com/

^4^https://www.oculus.com/go/

^5^https://www.oculus.com/quest/

^6^https://enterprise.vive.com/ca/vivefocus/

^7^https://www.pico-interactive.com/neo

^8^https://www.mi.com/global/mivr1c/

^9^https://www.vive.com/eu/wireless-adapter/
